# The estradiol-to-periovulatory follicle ratio (E2/N16) as a marker of follicular endocrine efficiency and embryological outcomes in ART cycles

**DOI:** 10.3389/fendo.2026.1866946

**Published:** 2026-08-21

**Authors:** Claudio Maurizio Maria Brigante, Ilaria Caliari, Roberta Iemmello, Silvana Gippone, Diletta Guglielmi, Beatrice Dal Canto, Mario Romano Mignini Renzini

**Affiliations:** Biogenesi Centro di Medicina della Riproduzione, Monza, Italy

**Keywords:** estradiol, periovulatory follicles, ovarian stimulation, embryo yield, assisted reproductive technology

## Abstract

**Background:**

Controlled ovarian stimulation (COS) in assisted reproductive technology (ART) is traditionally assessed using quantitative parameters, particularly the number of retrieved oocytes. However, these measures may not fully capture the endocrine efficiency of the periovulatory follicular cohort.

**Objective:**

To evaluate the association between the estradiol-to-periovulatory follicle ratio (E2/N16) and embryo yield, and to investigate its relationship with serum progesterone concentrations and ovarian stimulation characteristics.

**Methods:**

This retrospective observational cohort study included 2,831 ART cycles performed between January 2022 and February 2025. E2/N16 was calculated by dividing serum estradiol concentration by the number of periovulatory follicles (≥16 mm) on the day of trigger. The primary outcome was viable embryo yield per cycle. E2/N16 was analyzed as a continuous variable and by quartiles. Multivariable models were adjusted for female age, body mass index, anti-Müllerian hormone, number of retrieved oocytes, total FSH dose, and trigger type. Restricted cubic splines with formal tests of non-linearity and sensitivity analyses were also performed.

**Results:**

Viable embryo yield progressively decreased across increasing E2/N16 quartiles, from 2.09 to 1.77 embryos per cycle (p < 0.001). After multivariable adjustment, including trigger type, higher E2/N16 remained independently associated with lower embryo yield (β = −0.000218; 95% CI, −0.000427 to −0.000010; p = 0.040). The spline showed no statistically significant departure from linearity (P for non-linearity = 0.449), despite a modest initial rise followed by a decline. The association with serum progesterone was significantly non-linear (P for non-linearity = 0.009), with a U-shaped pattern. After exclusion of cycles with two or fewer periovulatory follicles, the association with embryo yield was attenuated but remained directionally consistent.

**Conclusions:**

The estradiol-to-periovulatory follicle ratio (E2/N16) was independently associated with embryo yield and provided information complementary to conventional quantitative measures of ovarian response. Its relationship with progesterone was non-monotonic, indicating that E2/N16 should not be interpreted as a simple linear marker of ovarian steroidogenesis. Prospective studies are required to validate its clinical utility and define how it might contribute to the assessment of ovarian stimulation in ART.

## Introduction

Controlled ovarian stimulation (COS) is a cornerstone of assisted reproductive technology (ART), aiming to increase the number of available oocytes and ultimately improve cumulative reproductive outcomes. Ovarian response is traditionally assessed using quantitative parameters, particularly the number of retrieved oocytes, which remains one of the strongest predictors of cumulative live birth following ART (1, 2).

However, quantitative measures alone do not fully characterize ovarian response. Increasing evidence indicates that reproductive outcomes depend not only on the number of retrieved oocytes but also on the biological characteristics of the recruited follicular cohort. Accordingly, both insufficient and excessive ovarian responses have been associated with less favorable reproductive outcomes, suggesting that follicular quantity alone does not completely explain treatment success (2–4).

Folliculogenesis is a highly coordinated process regulated by endocrine and intraovarian mechanisms that control follicular recruitment, selection, and growth (5). During COS, multiple follicles develop simultaneously but exhibit substantial biological heterogeneity. Consequently, follicles of similar size may differ in their endocrine activity and their ability to yield mature oocytes and viable embryos (6–10).

In routine clinical practice, serum estradiol (E2) concentrations and follicular size are the principal parameters used to monitor ovarian stimulation and determine the timing of ovulation triggering. Nevertheless, each provides only partial information. Serum estradiol reflects the overall steroidogenic activity of the developing follicular cohort but does not account for the number of follicles contributing to hormone production, whereas follicular diameter provides a morphological estimate of development without directly reflecting endocrine function.

To overcome these limitations, several estradiol-based indices have been proposed to better characterize ovarian stimulation. Previous studies have evaluated the estradiol-to-oocyte ratio and the estradiol-to-follicle ratio as indicators of stimulation efficiency and predictors of reproductive outcomes (11–15). Collectively, these studies suggest that integrating endocrine activity with follicular output provides clinically relevant information beyond absolute estradiol concentrations or follicle counts alone. However, these indices were derived from either the total follicular cohort or the number of retrieved oocytes and therefore do not specifically reflect the endocrine activity of the periovulatory follicular population.

Progesterone measured on the day of trigger represents another endocrine parameter commonly evaluated during ART cycles. Although elevated progesterone concentrations have primarily been associated with impaired endometrial receptivity and reduced pregnancy rates (16, 17), they also reflect the overall steroidogenic activity of the developing follicular cohort and may therefore provide complementary information on ovarian endocrine function.

Studies evaluating follicular size have consistently shown that follicles measuring approximately 16–22 mm on the day of trigger have the highest probability of yielding mature oocytes and favorable embryological outcomes (6–10). The interval between hCG administration and oocyte retrieval has also been investigated as a determinant of oocyte maturity and other ART outcomes (18). These observations provide the rationale for focusing specifically on the periovulatory follicular cohort rather than on the entire follicular population.

To our knowledge, no previous study has evaluated an estradiol-based index normalized to the number of periovulatory follicles (≥16 mm). We therefore hypothesized that relating serum estradiol concentrations to the number of periovulatory follicles could provide a clinically relevant estimate of follicular endocrine efficiency beyond indices based on the entire follicular cohort or on retrieved oocytes.

Accordingly, the aim of this study was to investigate the association between the estradiol-to-periovulatory follicle ratio (E2/N16), defined as the serum estradiol concentration divided by the number of follicles measuring ≥16 mm on the day of trigger, and embryo yield in ART cycles. We also explored its relationship with serum progesterone concentrations and ovarian stimulation characteristics. We hypothesized that progressively higher E2/N16 values reflect greater estradiol production relative to the size of the periovulatory follicular cohort and are associated with lower embryo yield.

## Materials and methods

### Study design and population

This retrospective observational cohort study included assisted reproductive technology (ART) cycles performed at Biogenesi – Centro di Medicina della Riproduzione (Monza, Italy) between January 2022 and February 2025.

**Figure 1:**
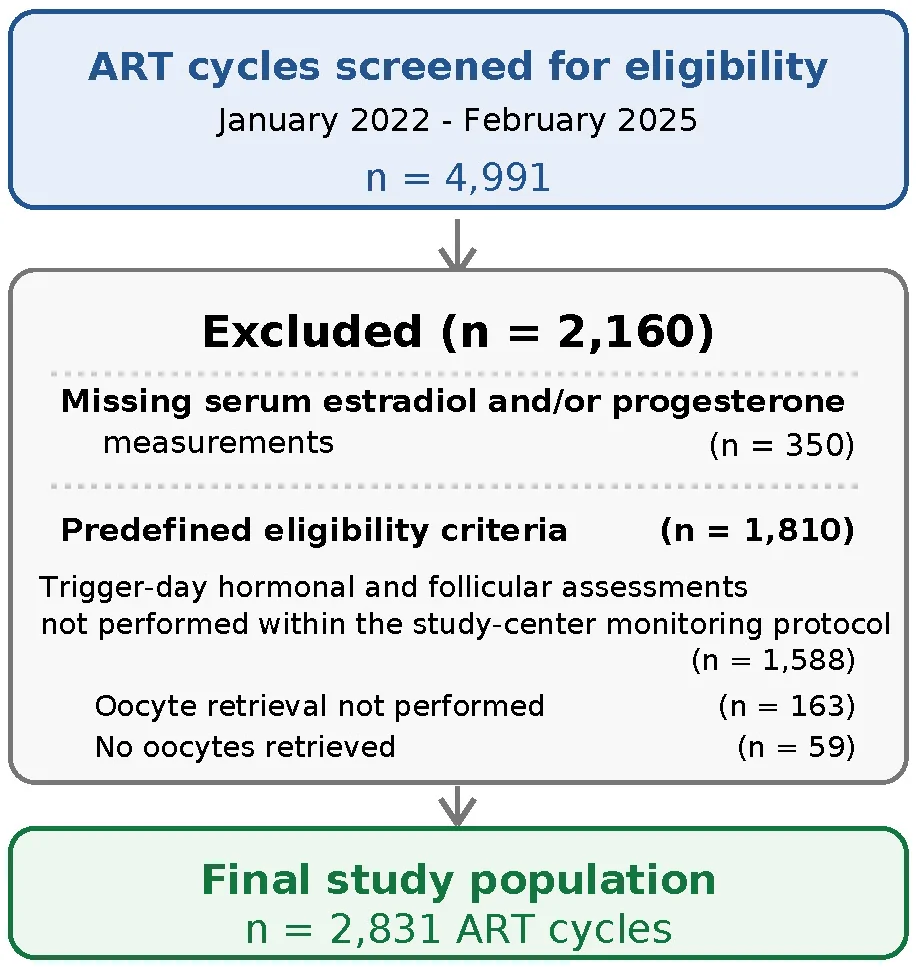
Study flow diagram of patient selection and final analytical cohort. A total of 4,991 ART cycles performed between January 2022 and February 2025 were screened for eligibility. Of 2,160 excluded cycles, 350 had missing trigger-day serum estradiol and/or progesterone measurements and 1,810 did not meet the predefined eligibility criteria, as itemized above. The final analytical cohort included 2,831 ART cycles.

All consecutive ovarian stimulation cycles performed during the study period were screened for eligibility (**Figure 1**). Overall, 4,991 ART cycles were identified. Of the 2,160 excluded cycles, 1,810 did not meet the predefined eligibility criteria: in 1,588 cycles, the paired trigger-day hormonal and follicular assessments required for E2/N16 calculation were not performed within the study-center monitoring protocol; oocyte retrieval was not performed in 163 cycles; and no oocytes were retrieved in 59 cycles. A further 350 otherwise eligible cycles were excluded because serum estradiol and/or progesterone measurements on the day of trigger were missing. The final study population comprised 2,831 ART cycles.

Eligible cycles required complete data on serum estradiol and progesterone concentrations measured on the day of trigger, follicular measurements allowing assessment of periovulatory follicles (≥16 mm), the number of retrieved and mature oocytes (MII), embryological outcomes, and anti-Müllerian hormone (AMH) levels. Cycles with no oocytes retrieved were excluded because the study focused on post-retrieval embryological efficiency and required an evaluable oocyte cohort.

To minimize potential confounding related to male-factor infertility, cycles using cryopreserved sperm or surgically retrieved sperm [e.g., testicular sperm extraction (TESE)] were excluded from analyses involving fertilization and embryo developmental outcomes.

### Ovarian stimulation, monitoring, and hormonal assessment

Controlled ovarian stimulation was performed using individualized gonadotropin regimens based on patient characteristics and ovarian response. Protocols included FSH alone, FSH combined with LH activity, and preparations containing both FSH and LH activity. Gonadotropin dose and treatment duration were individualized according to the treating physician’s clinical judgment.

Follicular development was monitored by serial transvaginal ultrasonography. Only follicular measurements obtained on the day of ovulation trigger were considered for the present analysis. Trigger timing was based on follicular development and hormonal parameters according to routine clinical practice.

Ovulation triggering was performed using recombinant hCG, GnRH agonist, or dual trigger according to ovarian response, follicular cohort characteristics, and the estimated risk of ovarian hyperstimulation syndrome.

Serum estradiol and progesterone concentrations were measured on the day of trigger using validated automated immunoassays performed on the Beckman Coulter platform (Beckman Coulter, Brea, CA, USA), routinely employed in the center. AMH values reported in pmol/L were converted to ng/mL using a conversion factor of 7.14. For quantitative analyses, trigger-day progesterone results reported below the assay quantification limit (n = 12) were assigned one-half of the stated limit.

### Exposure variable

Periovulatory follicles were defined as follicles measuring ≥16 mm in mean diameter on the day of trigger. This threshold was selected because follicles of this size have the highest probability of yielding mature oocytes and contributing to subsequent embryo development (6–10).

The primary exposure variable was the estradiol-to-periovulatory follicle ratio (E2/N16), calculated as serum estradiol concentration (pg/mL) divided by the number of periovulatory follicles (≥16 mm). E2/N16 was evaluated as a surrogate marker of follicular endocrine efficiency.

### Outcomes

The primary outcome was embryo yield, defined as the number of viable embryos obtained per cycle. Viable embryos included cleavage-stage embryos transferred or cryopreserved on days 2–3 and blastocysts cryopreserved on days 5–6.

Secondary embryological outcomes included the number of retrieved oocytes, mature oocytes (MII), mature oocyte rate (%MII), viable embryo-to-MII ratio, and total fertilization failure. Serum progesterone concentration on the day of trigger and total FSH dose administered during ovarian stimulation were evaluated as endocrine and stimulation-related variables, respectively.

### Statistical analysis

Continuous variables are presented as mean ± standard deviation (SD) or median (interquartile range [IQR]), as appropriate. Normality was assessed using the Shapiro–Wilk test.

The E2/N16 ratio was analyzed both as a continuous variable and after categorization into quartiles according to its distribution within the study population.

Each quartile represented approximately 25% of the study population: Q1, lowest 25% (≤255.1; n=708); Q2, 25th–50th percentile (>255.1 to ≤352.8; n=708); Q3, 50th–75th percentile (>352.8 to ≤469.7; n=708); and Q4, highest 25% (>469.7; n=707). These sample-derived boundaries were used for descriptive comparisons and were not intended as clinical cut-offs.

Comparisons among quartiles were performed using one-way analysis of variance (ANOVA) or the Kruskal–Wallis test, as appropriate. Pairwise comparisons were performed using the Mann–Whitney U test with Bonferroni correction when indicated. Associations between continuous variables were assessed using Spearman’s rank correlation coefficient.

The independent association between E2/N16 and embryo yield was evaluated using multivariable linear regression. The final model included female age, body mass index (BMI), anti-Müllerian hormone (AMH), number of retrieved oocytes, total FSH dose, E2/N16, and trigger type. Trigger type was entered as a categorical covariate (recombinant hCG as the reference category; GnRH agonist and dual trigger as indicator variables). Because antagonist protocols accounted for more than 80% of all cycles, stimulation protocol was not included owing to limited variability. Regression coefficients (β), heteroskedasticity-robust (HC3) standard errors, 95% confidence intervals (CI), and corresponding p values were reported.

Because embryo yield represents a count outcome, a negative binomial regression model was performed as a sensitivity analysis to account for potential over-dispersion. The model included the same covariates as the primary multivariable model, including trigger type. Incidence rate ratios (IRR), 95% confidence intervals (CI), and corresponding p values were reported.

Potential non-linear associations of E2/N16 with embryo yield and serum progesterone were examined using restricted cubic spline models with knots placed at the 5th, 35th, 65th, and 95th percentiles of the E2/N16 distribution. Spline models were adjusted for female age, BMI, AMH, number of retrieved oocytes, total FSH dose, and trigger type. Departure from linearity was assessed using a joint Wald test of the non-linear spline terms.

A sensitivity analysis was performed after excluding cycles with two or fewer periovulatory follicles (N16 ≤2) to evaluate the association between E2/N16 and embryo yield in cycles with a larger periovulatory follicular cohort. In addition, an interaction term between E2/N16 and the number of periovulatory follicles was included in the multivariable model to assess whether follicular cohort size modified the association.

Because of the retrospective observational design, no *a priori* sample size calculation was performed. All consecutive eligible ART cycles during the study period were included.

Statistical analyses were performed using IBM SPSS Statistics version 29.0 (IBM Corp., Armonk, NY, USA). All tests were two-sided, and *p* values <0.05 were considered statistically significant.

### Ethics statement

The study was conducted in accordance with the principles of the Declaration of Helsinki and applicable national regulations governing retrospective observational research. The analysis was based exclusively on retrospectively collected clinical data that had been fully anonymized before extraction and statistical analysis. According to applicable national regulations, retrospective studies based solely on anonymized clinical data do not require formal ethics committee approval or additional informed consent for research purposes. All data were handled in accordance with institutional policies and the General Data Protection Regulation (GDPR).

## Results

### Study population

The study selection process is shown in **Figure 1**. Between January 2022 and February 2025, 4,991 ART cycles were screened. Of 2,160 excluded cycles, 350 had missing serum estradiol and/or progesterone measurements on the day of trigger and 1,810 did not meet the predefined eligibility criteria. The latter comprised 1,588 cycles without paired trigger-day hormonal and follicular assessments performed within the study-center monitoring protocol, 163 cycles in which oocyte retrieval was not performed, and 59 cycles in which no oocytes were retrieved. The final analysis included 2,831 ART cycles. Baseline characteristics according to E2/N16 quartiles are presented in **Table 1**.

**Table 1:** Baseline characteristics, stimulation parameters, embryological outcomes, and hormonal profiles according to E2/N16 quartiles.

Variable	Quartile 1 (n = 708)	Quartile 2 (n = 708)	Quartile 3 (n = 708)	Quartile 4 (n = 707)	P-value
Baseline characteristics					
Female age, years (mean ± SD)	36.3 ± 4.1	36.7 ± 4.2	37.6 ± 4.0	37.5 ± 4.0	<0.001
Body mass index, kg/m² (mean ± SD)	23.15 ± 3.65	22.82 ± 3.52	22.45 ± 3.30	22.29 ± 3.36	<0.001
Anti-Müllerian hormone, ng/mL (median [IQR])	2.14 (1.13–3.77)	1.72 (0.89–3.16)	1.42 (0.79–2.72)	1.44 (0.81–2.60)	<0.001
Total FSH dose, IU (mean ± SD)	2122 ± 964	2308 ± 994	2484 ± 1015	2454 ± 973	<0.001
Number of periovulatory follicles (≥16 mm), mean ± SD	8.36 ± 5.15	7.11 ± 4.09	6.03 ± 3.28	4.70 ± 2.98	<0.001
Embryological outcomes					
Retrieved oocytes, mean ± SD	9.79 ± 7.13	9.38 ± 7.12	8.24 ± 5.78	8.49 ± 6.46	<0.001
Mature oocytes (MII), mean ± SD	6.27 ± 4.78	5.97 ± 4.59	5.24 ± 3.97	5.40 ± 4.18	<0.001
MII rate, %	64.1	63.6	63.6	63.6	0.90
Viable embryos, mean ± SD	2.09 ± 1.73	2.00 ± 1.60	1.81 ± 1.45	1.77 ± 1.51	<0.001
Viable embryos/MII, %	33.3	33.4	34.5	32.9	0.34
Hormonal parameters					
Serum progesterone on the day of trigger, ng/mL (median [IQR])	1.00 (0.70–1.50)	1.00 (0.70–1.50)	1.00 (0.70–1.50)	1.10 (0.80–1.60)	<0.001

Values are presented as mean ± standard deviation (SD) or median [interquartile range (IQR)], as appropriate. E2/N16 was calculated as serum estradiol concentration (pg/mL) divided by the number of periovulatory follicles (≥16 mm) on the day of trigger. p values refer to comparisons across quartiles using one-way analysis of variance or the Kruskal–Wallis test, as appropriate. Progesterone results below the assay quantification limit (n = 12) were assigned one-half of the stated limit.

AMH, anti-Müllerian hormone; E2/N16, estradiol-to-periovulatory follicle ratio; FSH, follicle-stimulating hormone; MII, metaphase II oocytes.

### Baseline characteristics and embryological outcomes

Baseline characteristics according to E2/N16 quartiles are summarized in **Table 1**. Women in the higher E2/N16 quartiles were older, had lower AMH concentrations, received higher total FSH doses, and developed fewer periovulatory follicles (all *p* < 0.01).

Embryological outcomes varied across quartiles. The mean number of retrieved oocytes decreased from 9.79 in Quartile 1 to 8.49 in Quartile 4 (*p* < 0.001), while the mean number of mature oocytes declined from 6.27 to 5.40 (*p* < 0.001).

**Figure 2:**
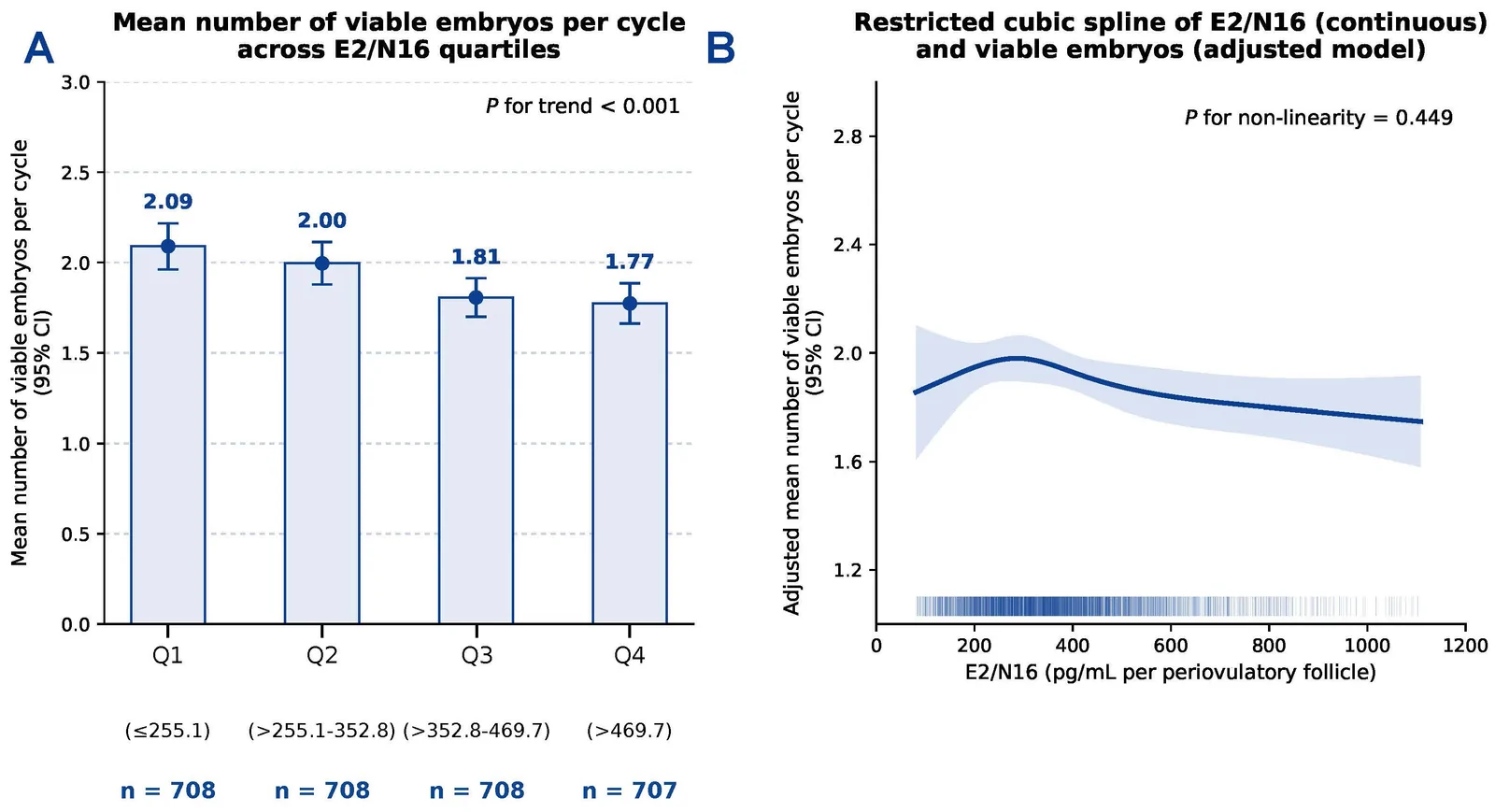
Association between E2/N16 and viable embryo yield. **(A)** Mean number of viable embryos per cycle (±95% confidence interval) according to E2/N16 quartiles. Numbers below the x-axis indicate the number of cycles in each quartile; the trend test treated quartiles as an ordinal variable. **(B)** Adjusted restricted cubic spline for continuous E2/N16 and viable embryo yield. The model was adjusted for female age, body mass index, anti-Müllerian hormone, number of retrieved oocytes, total FSH dose, and trigger type. The shaded area indicates the 95% confidence interval; rug marks show the E2/N16 distribution. E2, estradiol; N16, number of periovulatory follicles ≥16 mm; CI, confidence interval; FSH, follicle-stimulating hormone.

Embryo yield also progressively decreased across increasing E2/N16 quartiles, from 2.09 embryos per cycle in Quartile 1 to 1.77 in Quartile 4 (p <0.001) (**Figure 2A**).

In contrast, neither the mature oocyte rate (%MII; p =0.90) nor the viable embryo-to-MII ratio (p =0.34) differed significantly among quartiles.

### Multivariable analysis

After adjustment for female age, body mass index (BMI), anti-Müllerian hormone (AMH), number of retrieved oocytes, total FSH dose, and trigger type, higher E2/N16 remained independently associated with lower embryo yield (β = −0.000218, 95% CI −0.000427 to −0.000010; p = 0.040) (**Table 2**).

**Table 2:** Multivariable analysis of factors independently associated with embryo yield.

Variable	β coefficient	Robust SE	95% CI	P value
E2/N16 ratio (per 1 pg/mL/follicle)	−0.000218	0.000107	−0.000427 to −0.000010	0.040
Female age (years)	−0.028	0.008	−0.043 to −0.013	<0.001
Body mass index (kg/m²)	−0.017	0.007	−0.031 to −0.002	0.023
Anti-Müllerian hormone (ng/mL)	0.009	0.013	−0.015 to 0.034	0.459
Retrieved oocytes (n)	0.094	0.007	0.079 to 0.108	<0.001
Total FSH dose (IU)	−0.000119	0.000027	−0.000172 to −0.000066	<0.001
GnRH agonist trigger vs hCG	−0.015	0.092	−0.195 to 0.166	0.873
Dual trigger vs hCG	0.078	0.083	−0.084 to 0.240	0.344
Intercept	2.828	0.351	2.140 to 3.516	<0.001

Values are reported as regression coefficients (β), heteroskedasticity-robust (HC3) standard errors (SE), 95% confidence intervals (CI), and p values. The model included all 2,831 cycles and was adjusted for female age, body mass index, anti-Müllerian hormone, number of retrieved oocytes, total FSH dose, E2/N16, and trigger type. Recombinant hCG was the reference category for trigger type.

AMH, anti-Müllerian hormone; CI, confidence interval; E2/N16, estradiol-to-periovulatory follicle ratio; FSH, follicle-stimulating hormone; SE, standard error.

Female age, BMI, and total FSH dose were independently associated with lower embryo yield, whereas the number of retrieved oocytes was positively associated with embryo yield. AMH and trigger type were not independently associated with embryo yield.

The restricted cubic spline showed a modest increase in adjusted embryo yield at the lower end of the E2/N16 distribution, followed by a decline at higher values (**Figure 2B**). However, the formal test did not support a statistically significant departure from linearity (P for non-linearity = 0.449); therefore, the apparent initial curvature should not be interpreted as evidence of a threshold or an inverted-U relationship.

Given the count distribution of embryo yield, a negative binomial regression analysis was performed as a sensitivity analysis. This model confirmed the inverse association between E2/N16 and embryo yield after adjustment for trigger type and the other covariates (IRR per 100-unit increase: 0.988, 95% CI 0.978–0.999; p = 0.040).

### Sensitivity analysis

In the sensitivity analysis excluding cycles with two or fewer periovulatory follicles (N16 ≤2), the association between E2/N16 and embryo yield was attenuated and did not reach statistical significance (β = −0.000228, 95% CI −0.000564 to 0.000107; p = 0.182). Nevertheless, the direction and magnitude of the estimated effect remained similar to those in the primary analysis.

To further assess whether follicular cohort size modified the association between E2/N16 and embryo yield, an interaction term between E2/N16 and the number of periovulatory follicles was included in the trigger-adjusted multivariable model. No significant interaction was observed (P for interaction = 0.708).

### Progesterone analysis

Serum progesterone concentrations showed a weak but statistically significant positive correlation with E2/N16 (Spearman’s ρ =0.077, p <0.001).

**Figure 3:**
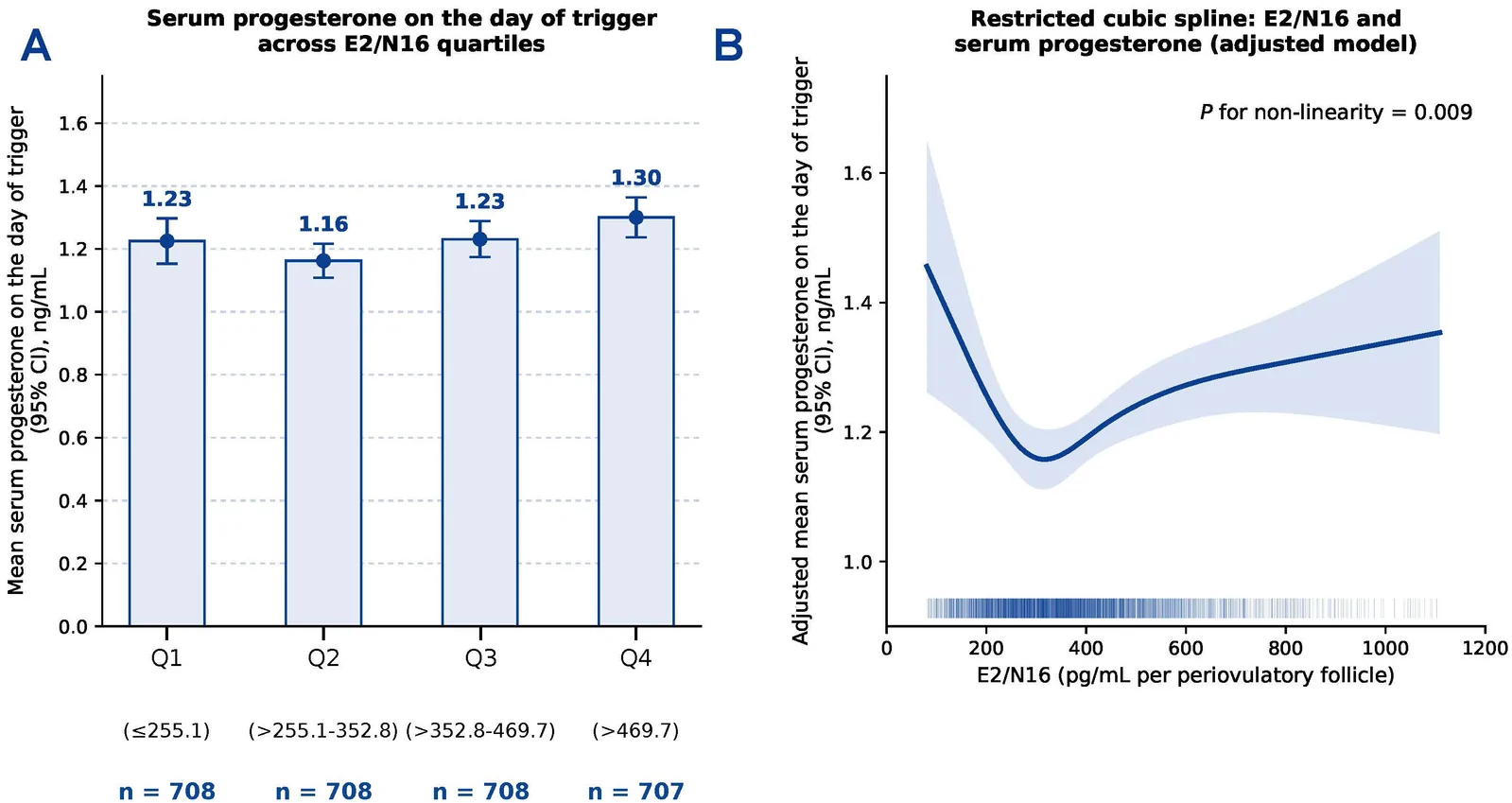
Association between E2/N16 and serum progesterone on the day of trigger. **(A)** Mean serum progesterone concentration on the day of trigger (±95% confidence interval) according to E2/N16 quartiles. All 2,831 cycles contributed to the progesterone analysis. Values reported below the assay quantification limit were assigned one-half of the stated limit. **(B)** Adjusted restricted cubic spline for continuous E2/N16 and serum progesterone. The model was adjusted for female age, body mass index, anti-Müllerian hormone, number of retrieved oocytes, total FSH dose, and trigger type. The shaded area indicates the 95% confidence interval; rug marks show the E2/N16 distribution. E2, estradiol; N16, number of periovulatory follicles ≥16 mm; CI, confidence interval; FSH, follicle-stimulating hormone.

Serum progesterone concentrations differed across E2/N16 quartiles and were highest in Quartile 4: median values were 1.00 (0.70–1.50), 1.00 (0.70–1.50), 1.00 (0.70–1.50), and 1.10 (0.80–1.60) ng/mL from Quartile 1 to Quartile 4, respectively (p <0.001) (**Figure 3A**). All 2,831 cycles contributed to this analysis.

After adjustment for female age, BMI, AMH, number of retrieved oocytes, total FSH dose, and trigger type, the simple linear coefficient for E2/N16 was not significant (β = 0.000087, 95% CI −0.000094 to 0.000269; p = 0.345). The restricted cubic spline demonstrated a significant non-linear association (P for non-linearity = 0.009) (**Figure 3B**). Progesterone concentrations were higher at the lowest E2/N16 values, decreased toward an approximate nadir around 300 pg/mL per periovulatory follicle, and subsequently increased at higher E2/N16 values, producing a U-shaped pattern.

## Discussion

The present study evaluated the estradiol-to-periovulatory follicle ratio (E2/N16) as a marker of follicular endocrine efficiency in ART cycles. The principal finding was a progressive decline in embryo yield across increasing E2/N16 quartiles. E2/N16 remained independently associated with embryo yield after adjustment for female age, BMI, AMH, the number of retrieved oocytes, total FSH dose, and trigger type. Although the spline displayed a modest rise at the lower end followed by a decline, the formal test of non-linearity was not significant, and the visual curvature should therefore not be interpreted as a validated threshold. The association was attenuated after exclusion of cycles with two or fewer periovulatory follicles but remained directionally consistent. Together, these findings suggest that E2/N16 provides information complementary to conventional quantitative measures of ovarian response.

Ovarian response has traditionally been evaluated according to the number of retrieved oocytes, which remains one of the strongest predictors of cumulative live birth after ART (1, 2). However, accumulating evidence indicates that reproductive outcomes depend not only on the quantity of oocytes retrieved but also on the biological characteristics of the recruited follicular cohort (2–4). These observations support the concept that quantitative measures alone do not fully characterize ovarian response. Our findings are consistent with this perspective and suggest that integrating endocrine activity with the size of the periovulatory follicular cohort may provide additional information beyond conventional markers of ovarian response.

Several estradiol-based indices have previously been proposed to assess ovarian stimulation. Orvieto et al. introduced the estradiol-to-follicle and estradiol-to-oocyte ratios as indicators of stimulation efficiency (11). Vaughan et al. subsequently reported an association between the estradiol-to-oocyte ratio and reproductive outcomes in a large IVF population (12), whereas Huang et al. demonstrated that lower estradiol production relative to follicle number was associated with poorer treatment outcomes (13). More recently, Chen et al. related the estradiol-to-follicle ratio to the subsequent risk of gestational diabetes after fresh embryo transfer (14), while Salović et al. evaluated the prognostic value of the estradiol-to-oocyte ratio in women with polycystic ovary syndrome (15). Collectively, these studies indicate that indices integrating endocrine activity with follicular output provide clinically relevant information beyond serum estradiol concentrations alone.

The present study extends these observations by focusing specifically on the periovulatory follicular cohort. Follicles measuring ≥16 mm on the day of trigger have the highest probability of yielding mature oocytes and contributing to subsequent embryo development (6–10). By normalizing serum estradiol concentrations to the number of these follicles, E2/N16 was designed to estimate the endocrine performance of the follicular cohort most directly involved in oocyte retrieval. In this context, follicular endocrine efficiency refers to the relationship between circulating estradiol production and the size of the periovulatory follicular cohort, rather than to steroidogenic activity alone. Persistence of the association after adjustment for ovarian reserve, ovarian response, stimulation intensity, female characteristics, and trigger type suggests that E2/N16 captures information not fully explained by conventional clinical markers.

AMH progressively decreased across increasing E2/N16 quartiles, indicating that ovarian reserve contributes, at least in part, to variation in this index. However, the differences between the upper quartiles were relatively modest and are unlikely to fully account for the observed reduction in embryo yield. Moreover, E2/N16 remained independently associated with embryo yield after multivariable adjustment, whereas AMH did not, supporting the hypothesis that the index reflects a dimension of ovarian response beyond ovarian reserve alone.

From a physiological perspective, serum estradiol reflects the overall steroidogenic activity of the developing follicular cohort. During controlled ovarian stimulation, multiple growing follicles contribute to circulating estradiol concentrations. Nevertheless, greater estradiol production relative to the number of periovulatory follicles should not be interpreted as evidence of greater follicular efficiency. Rather, E2/N16 represents a surrogate marker integrating endocrine output with the size of the follicular cohort reaching the periovulatory stage. The biological mechanisms underlying the inverse association with embryo yield remain to be elucidated.

Interestingly, higher E2/N16 values were associated with lower embryo yield despite similar mature oocyte rates and viable embryo-to-MII ratios across quartiles. Although the present study was not designed to investigate the mechanisms responsible for this association, these findings suggest that E2/N16 is unlikely to reflect differences in oocyte maturation alone. Instead, it may identify variation in the endocrine characteristics of the periovulatory follicular cohort that is not captured by conventional embryological indicators.

The relationship between E2/N16 and progesterone was more complex than suggested by quartile summaries or a single linear coefficient. After adjustment for trigger type and the other covariates, the linear coefficient was no longer significant, whereas the spline demonstrated a significant U-shaped association. Progesterone was higher at very low E2/N16 values, decreased toward an approximate nadir around 300 pg/mL per periovulatory follicle, and increased thereafter. This pattern suggests that low E2/N16 values should not automatically be interpreted as a uniformly favorable endocrine state and may reflect a different endocrine pattern at the lower end of the distribution. Conversely, the rise at higher E2/N16 values is consistent with increasing progesterone production in cycles with greater estradiol output relative to the periovulatory follicular cohort. Given the recognized association between trigger-day progesterone elevation and endometrial receptivity (16, 17), both extremes may have potential clinical relevance. However, the present study did not assess fresh-transfer eligibility or define clinical thresholds; the approximate nadir should therefore not be interpreted as a validated cut-off.

The sensitivity analysis attenuated the association between E2/N16 and embryo yield after exclusion of cycles with two or fewer periovulatory follicles, although the estimated coefficient remained similar in direction and magnitude. Moreover, no significant interaction was observed between E2/N16 and the number of periovulatory follicles in the trigger-adjusted model. These findings provide no evidence that the association differed materially according to follicular cohort size, but the reduced precision in the restricted cohort warrants cautious interpretation and prospective confirmation.

This study has several limitations. First, its retrospective observational design precludes causal inference, and residual confounding, including confounding by indication in the choice of trigger, cannot be excluded despite multivariable adjustment. Second, the exclusion of cycles lacking standardized paired trigger-day assessments or complete hormonal data may have introduced selection bias and may limit generalizability. Third, embryo developmental competence was evaluated using embryo yield, whereas detailed embryo quality parameters, including morphological grading and blastulation rate, were not analyzed as secondary outcomes. Clinical pregnancy and live-birth outcomes were also not evaluated because the study was cycle-based and included heterogeneous fresh and freeze-all strategies with subsequent frozen embryo transfers. Fourth, no *a priori* sample size calculation was performed because of the retrospective observational design. Finally, the biological mechanisms underlying the observed associations remain speculative. Despite these limitations, E2/N16 may capture an additional dimension of ovarian response not represented by conventional quantitative parameters alone. Future prospective studies incorporating detailed embryological, transfer, and cumulative reproductive outcomes are needed before the index can be used to guide trigger timing or stimulation strategies.

## Conclusion

The present study indicates that the estradiol-to-periovulatory follicle ratio (E2/N16) is independently associated with embryo yield in ART cycles after adjustment for major patient- and treatment-related factors, including trigger type. By integrating serum estradiol concentration with the number of periovulatory follicles, E2/N16 provides information complementary to conventional quantitative measures of ovarian response.

The significant non-linear relationship between E2/N16 and serum progesterone indicates that endocrine activity relative to the periovulatory follicular cohort is not adequately represented by a simple monotonic association. The U-shaped pattern should be considered exploratory and does not establish a clinical threshold.

Although E2/N16 is simple and clinically accessible, prospective validation is required before it can be incorporated into clinical decision-making. Future studies should determine whether it improves the assessment of ovarian stimulation and whether either extreme of its distribution has reproducible implications for embryological or cumulative reproductive outcomes.

## Data Availability

The raw data supporting the conclusions of this article will be made available by the authors, without undue reservation.
